# Development of Stability-Indicating Methods for Cefquinome Sulphate

**Published:** 2013-09

**Authors:** Shaza W. Shantier, Elrasheed A. Gadkariem, Mohamed O. Adam, Magdi A. Mohamed

**Affiliations:** Department of Pharmaceutical Chemistry, Faculty of Pharmacy, University of Khartoum, Sudan

**Keywords:** cefquinome sulphate, derivative spectrophotometry, HPLC, kinetics, suspension

## Abstract

The degradation behavior of cefquinome sulphate in alkaline medium at different temperatures was investigated using both first derivative spectrophotometric and HPLC methods. The drug degradation was found to be pH and temperature dependant. The pH-rate profile indicated a first order dependence of K_obs_ on [OH^-^] at pHs ranging between 9 and 11. Arrhenius plot obtained at pH 10 was linear between 65° and 100°C. The estimated activation energy of the hydrolysis was found to be 21.1 kcal mol^-1^. Stability-indicating thin-layer chromatographic method for the separation of the drug and its alkaline hydrolysis product has been developed.

## INTRODUCTION

The need to develop a stability-indicating method using stress degradation has been recommended by International Conference of Harmonization (1). In practice, the effects of pH and temperature changes on drug stability are often used in such studies. The results of such studies are of great importance in the estimation of drug shelf life and the effect of degradation products on decreasing efficacy and causing toxicity. They may also serve as guides for better drug design, drug formulation and drug analysis.

**Figure 1:**
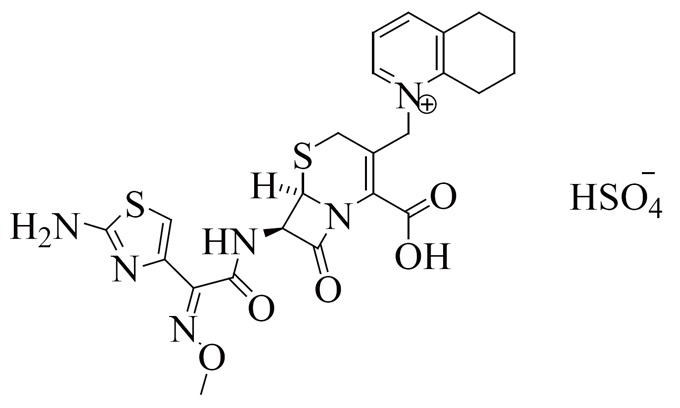
Chemical structure of cefquinome sulphate.

Cefquinome Sulphate (CS) is Quinolinium (Fig. 1) (2): 1-[[(6R,7R)-7-[[(2Z)-(2-amino-4-thiazolyl)(methoxyimino)acetyl]amino]-2-carboxy-8-oxo-5-thia-1-azabicyclo[4.2.0]oct-2-en-3-yl]methyl]-5,6,7,8-tetrahydro-,sulfate. CS has a broad spectrum of activity against both Gram- positive and Gram-negative bacteria including *Pseudomonas aeruginosa.* It is usedfor the treatment of bovine respiratory disease (BRD) (3).

Literature review revealed that no previus spectrophotometric or chromatographic methods have been developed for the stability studies on CS in its bulk or pharmaceutical dosage form (4-8).

Therefore, the aim of the present work was to investigate the effects of pH, temperature and alkali on the stability of CS in bulk and dosage form using developed stability indicating methods.

## EXPERIMENTAL

### Instrumentation

UV spectrophotometric studies were carried out on Shimadzu UV-1800ENG240V, (Koyoto, Japan).

HPLC system consisted of a Shimadzu liquid chromatograph using Shimpack column VP-ODS (25 × 4.6 mm). The system was equipped with a UV-visible detector (SPD-20A prominence), P/N 7725i injector, 20 μL loop, DGU-20A3 prominence degasser and LC-20 AB pump. Thin-layer chromatography (TLC) was conducted on precoated silica gel sheets 60F254, 5 × 10 cm with 0.2 mm thickness.

### Chromatographic conditions

Chromatographic separation was achieved using mobile phase consisting of ammonium acetate buffer: acetonitrile (80:20 v/v). The buffer solution was filtered through 0.45 µm membrane filter. The mobile phase was pumped through the column at a flow rate of 1 ml min^-1^.UV detection was performed at 268 nm and 20 µl volumes were injected onto the column at room temperature.

The solvent systems used in TLC were: methanol: ethylacetate: acetone: water (5:2.5:2.5:1.5 v/v) and ammonium acetate pH (6.2): acetonitrile (85:15 v/v). Visualization was accomplished under UV light (254 nm).

### Reference sample and reagents

A drug sample of CS (Cobactan^®^ 2.5%) was kindly provided by Intervet Schering-Plough, European Union.The reference standard, certified to contain 99.8%, was provided by Intervet International GmbH. Disodium orthophosphate (BDH) Limited Poole, England; Sodium hydroxide (BDH), Pool, England; Acetonitrile (Lobachemie, India); Ammonium acetate (E. Merck, Germany). All other chemicals used were of analytical grade reagents.

Phosphate buffer (pH range 2.2- 8) (9), phosphate buffer (pH range 9-11) (10), borate buffer (pH range 8-10) (11) and ammonium acetate 15% (pH adjusted to 6.2 using glacial acetic acid) were prepared.

### Sample solution

CS sample solution (1000 μg/ml) was freshly prepared in distilled water. After filteration, the solution was further diluted to obtain 20µg/ml solution (solution A).

### Procedures

**Effect of pH on the stability of CS solution.** Aliquots from solution A (3 ml) were transferred to 11 stoppered glass tubes. 1ml of the phosphate buffer (pH values 2.2, 3, 4, 5, 6, 7, 7.4, 8, 9, 10 and 11) was added to each tube. The solutions were heated in boiling water bath for 30 minutes. The reaction was then quenched by cooling, and the volumes were completed to 10ml with distilled water. The kinetics of the decomposition of the drug was monitored by first derivative spectrophotometry scanning between 400nm and 240nm. The rate constant for each reaction mixture was calculated from the plot of log [remaining drug] *vs* time (12).

**Effect of alkali on the stability of CS solution.** Aliquots from solution A (3 ml) were transferred to five stoppered glass tubes. 1 ml of sodium hydroxide solution (1 M) was added to each tube. The volume of one glass tube was completed to 10ml with distilled water and the first derivative spectrum of the solution was then recorded. The other four stoppered glass tubes were heated in a boiling water bath at suitable time intervals (15, 30, 45 and 60 minutes). The reaction was quenched by cooling. The volumes were then completed to 10ml with distilled water. The solutions were monitored by the first derivative spectrophotometry (240-400 nm) and HPLC method. The same procedure was repeated using 0.5 M and 2 M sodium hydroxide solution instead of 1 M to study the effect of sodium hydroxide concentration.

**Effect of temperature on the stability of CS solution.** Aliquots from solution A (3 ml) were transferred to four stoppered glass tubes. 1 ml of phosphate buffer (pH 10) was added to each tube. The solutions were then heated at the appropriate temperature of the study (65º and 80ºC) with the appropriate time intervals ranging between 10-40 minutes. The reaction was then quenched by cooling and the volumes were completed to 10 ml with distilled water. The kinetics of the decomposition was monitored using first derivative spectrophotometry.

**Thin-layer chromatographic method.** Stock solution of CS (1 mg/ml) was freshly prepared. 3 ml of the solution was transferred to stoppered glass tube. 1ml of 1 M sodium hydroxide was added, and the solution was heated in a boiling water bath for 30 minutes. The reaction was then quenched by cooling, acidified with hydrochloric acid (1.3 ml, 1 M). The TLC plate has been spotted with the hydrolysed and unhydrolysed CS solution. The plate was then run using the selected mobile phase, dried and observed under the UV-light (254 nm).

## RESULTS AND DISCUSSION

The stability of a pharmaceutical product is defined as the capability of the product to retain its efficacy, properties and characteristics throughout its shelf life (13). One of the most important types of stability is chemical stability which includes hydrolysis. Amides are generally more stable to hydrolysis than esters. In general, the rate of hydroxyl ion- catalyzed reaction of amides is greater than the rate of proton-catalyzed hydrolysis (14).

Cephalosporines are amides in which the amide bond is part of strained four-membered β-lactam ring. The decomposition of these compounds is catalyzed by solvent, hydroxide ion, and many buffer species and thus is unstable to be formulated as solutions (13, 15).

### Effect of pH on the stability of CS solution

**Figure 2:**
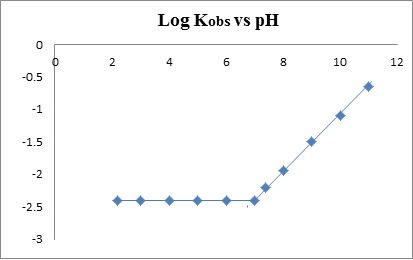
pH-rate profile for the degradation of CS at 100°C (pHs 2.2-11).

The decompostion of CS was monitored by the first derivative spectroscopy over the pH range 2.2-11. A plot of log K_obs_ vs pHs gave a slope value of +0.43 (r=0.9994) on the alkaline side. This positive value suggests a first order dependence of the observed rate constant (K_obs_) on [OH^-^]. The pH-profile plot obtained (Fig. 2) resembles subtype BCD in the generalized pH-profile polygon (12), where the magnitude of (K_obs_) increased rapidly at higher pH values and it is also reflected by the short t 1/2 values obtained (Table 1).

**Table 1:** Kobs, t_½_ and t_90_ values for the degradation of cefquinome at 100°C in phosphate buffer at different pHs

pH	K_obs_ min^-1^	t_½_ min	t _90_

11	0.224	3.09	0.468
10	0.079	8.77	1.33
9	0.032	21.66	3.28
8	0.011	63	9.55
6-7	0.004	173.25	26.25

These findings suggest that the formulation of this drug in liquid form should be at pHs on the acidic side (2-6) with a medium value between 4-5.

### UV spectral changes in alkali-treated CS solution

**Figure 3:**
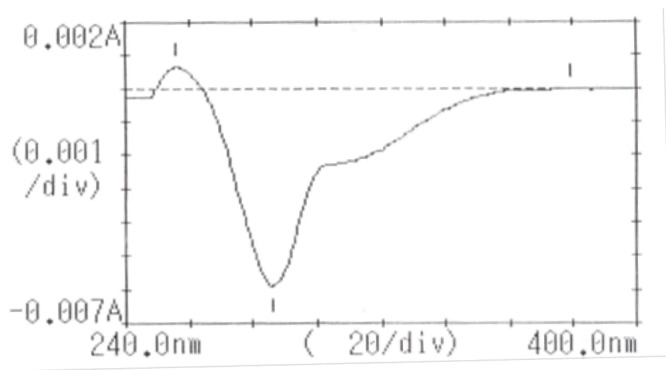
First derivative spectrum of CS solution (8 μg/ml, 286 nm).

**Figure 4:**
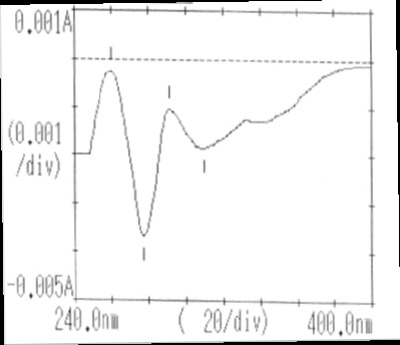
First order derivative of the degraded CS (heating time 30 min).

The first derivative spectrum of CS solution shown in (Fig. 3) exhibited a major peak at 286 nm. Treatment of this solution with alkali resulted in the reduction of its absorption peak and appearance of another peak at 311 nm which seems to be an alkaline hydrolysis product of CS (Fig. 4). This shift in wavelength maxima suggests a product of more conjugation than the parent compound.

### Effect of alkali on the stability of CS solution

The effect of different sodium hydroxide concentrations coupled with different heating time intervals on CS solution degradation rate was studied. The most suitable heating time interval that can give measurable degradation rate with good correlation coefficient was found to be 15 minutes interval. The alkali effect was studied using first derivative spectrophotometric and HPLC methods. The first derivative spectral changes of CS solution treated with 1 M sodium hydroxide at boiling water bath, reflect a decrease in CS peak at its λ_max_ 286 nm and consequent formation of degradation product with λ_max_ 311 nm (Fig. 4).

The degradation rate constant was calculated by using linear regression analysis data of log % remaining vs time for three time intervals Table 2. The degradation rate was increased, and subsequently the t½ decreased, with increasing sodium hydroxide concentration.

**Table 2:** Effect of 1 M sodium hydroxide on the stability of CS

Time (min)	Absorbance (cm)		Mean	% Remained	Log %

0	4.2	4.0	4.1	100	2
15	3.8	3.6	3.7	90.2	1.955
30	2.5	2.3	2.4	58.5	1.767
45	1.9	1.8	1.85	45	1.65

**Figure 5:**
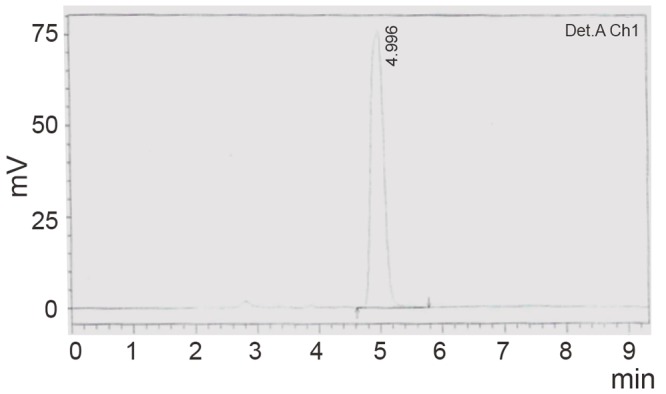
Typical chromatogram for the intact drug.

**Figure 6:**
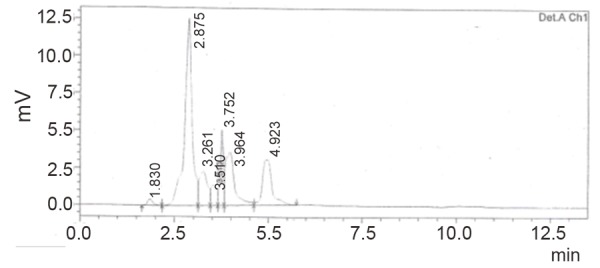
Typical chromatogram for degraded CS with 1 M NaOH (heating time 30 minutes).

Using the HPLC method, CS eluted at retention time of about 5 minutes (Fig. 5). Fig. 6 reflects a typical chromatogram of degraded CS solution after heating for 30 minutes with 1 M sodium hydroxide. The peak of the parent compound reflected a decrease in its concentration with subsequent appearance of a major peak at retention time 2.8. This peak was probably due to the alkaline hydrolysis product which has λ_max_ at 311 nm on the first derivative spectra.

The other small peaks appearing in the chromatogram suggest that CS undergoes a number of minor alkaline hydrolysis products beside the major one. This is in agreement with the previous reports (16).

### Effect of temperature on rate of hydrolysis of CS

**Figure 7:**
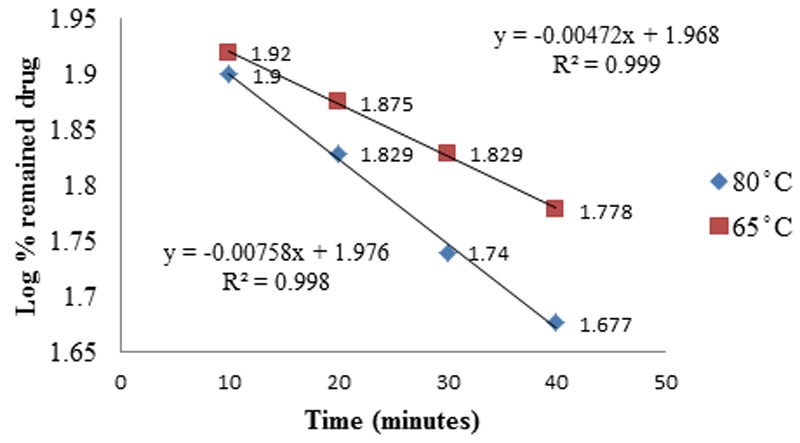
Time-course of the decomposition of cefquinome in phosphate buffer pH10 at different temperatures.

**Figure 8:**
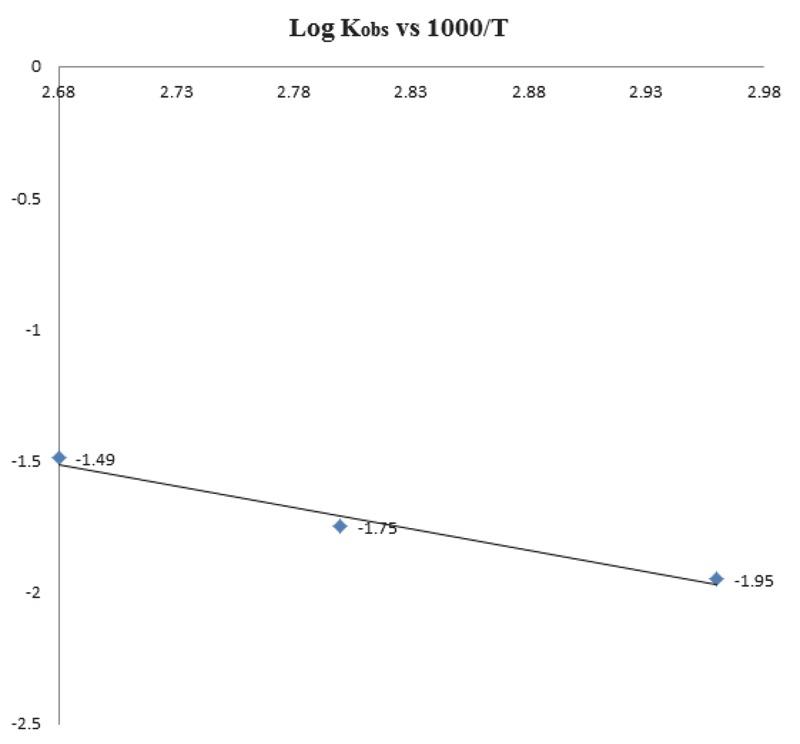
Arrhenius plot for degradation of cefquinome in phosphate buffer pH 10 and temperatures 65, 80 and 100°C.

The influence of temperature (65°, 80°) on the hydrolysis rate was studied by the first derivative spectrophotometry using phosphate buffer of pH 10. The hydrolysis process followed first-order kinetics at all temperatures (Fig. 7). Using the Arrhenius plot (Fig. 8), the calculated activation energy (Ea) was 21.1 kcal mol^-1^, which lies within the typical limits for hydrolysis reaction (17). The calculated Ea was utilized to calculate the shelf-life of CS (t_90_) and t½ (Table 3).

**Table 3:** K, t_½_ and t_90_ values for CS degradation at pH 10 at different temperatures

Temp.	K (min^-1^)	t_½_ (0.693/K)	t_90_ (0.105/K)

5°C	0.00186	372.58	56.45
25°C	0.00339	204.4	30.97
65°C	0.0112	61.88	9.375
80°C	0.0176	39.38	5.97
100°C	0.024	28.88	4.475

### TLC method

In order to obtain a suitable solvent for the separation of CS and its degradation product, mixtures of solvents with different polarity and percentages were investigated. The most appropriate mobile phase consisted of ammonium acetate acetonitrile (85:15) with a running time of about 15 minutes for 7 cm distance. This solvent successfully separated the drug and its major degradation product. The R_f_ values were 0.47 and 0.29 respectively.

### Effect of light on stability of CS

The light effect on CS was also studied. The study was carried at sunlight and UV irradition (254nm) in quartz cell and glass bottles using different solvent (water and methanol). CS solution was found to be stable under these conditions.

### Proposed scheme for the alkaline hydrolysis of CS

**Figure 9:**
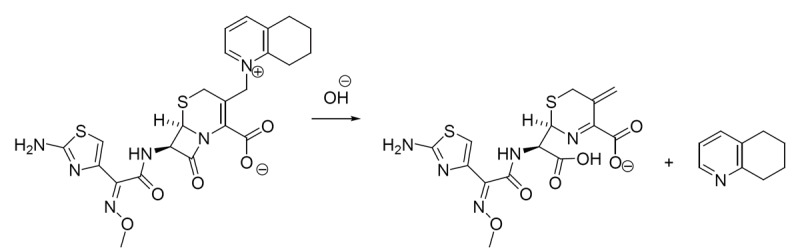
Proposed pathway for cefquinome degradation.

Cephalosporines are well known to degrade in alkaline media to give penicilloic acid (18). In this study, degradation rate was found to be [OH^-^] dependant, suggesting that the OH^-^ is playing the role of nucleophile (intramolecular hydrolysis) to give the proposed degradation product (Fig. 9).

The proposed degradation product absorbs at lower wavelength (311 nm) than that which was proposed for some other cephalosporines (19). The hydrolysis of those cephalosporines was affected by the presence of the free α-amino function, producing the diketopiperazine derivative which absorbs at 340 nm. Since CS lacks the amino group, it was suggested to undergo the intramolecular hydrolysis.

## CONCLUSION

The results of this study showed that CS solution is degraded via a hydrolysis process, which appears to be temperature and [OH^-^] dependant.

The results obtained reflect that the degradation rate, and subsequently the t½, decreases with increasing sodium hydroxide concentration (ranging from 0.5 M TO 2 M). CS was found to be stable along the acidic pH (2-6) even at temperatures above 80°C.

The effect of different buffer species (borate buffer) was also studied, which revealed no significant difference from the phosphate buffer.

The HPLC and TLC methods developed in this study were successfully used to separate the alkaline degradation products from the parent compound.
